# Polyglycerols as Multi-Functional Platforms: Synthesis and Biomedical Applications †

**DOI:** 10.3390/polym14132684

**Published:** 2022-06-30

**Authors:** Paria Pouyan, Mariam Cherri, Rainer Haag

**Affiliations:** Institute of Chemistry and Biochemistry, Freie Universität Berlin, Takustr. 3, 14195 Berlin, Germany; paria.pouyan@fu-berlin.de (P.P.); mcherri6@zedat.fu-berlin.de (M.C.)

**Keywords:** linear polyglycerol, hyperbranched polyglycerol, ring-opening polymerization of glycidol

## Abstract

The remarkable and unique characteristics of polyglycerols (PG) have made them an attractive candidate for many applications in the biomedical and pharmaceutical fields. The presence of multiple hydroxy groups on the flexible polyether backbone not only enables the further modification of the PG structure but also makes the polymer highly water-soluble and results in excellent biocompatibility. In this review, the polymerization routes leading to PG with different architectures are discussed. Moreover, we discuss the role of these polymers in different biomedical applications such as drug delivery systems, protein conjugation, and surface modification.

## 1. Introduction

Since the introduction of “macromolecules” in the 1920′s by Hermann Staudinger, the field of chemistry has been tremendously transformed, and the resulting polymer science has revolutionized modern life in many different aspects, especially in life sciences and medicine. The chemical foundation of polymers has led to the design of an endless variety of polymeric structures with different physical and chemical properties [1]. The immense impact of polymers on the field of medicine and pharmaceuticals has resulted in the development of many advanced biomaterials for prevalent applications such as carriers in smart drug delivery [2], multivalent antiviral agents [3,4], and anti-inflammatory [5] agents. Biocompatibility, water-solubility, and non-toxicity are the requirements for polymers in biomedical and pharmaceutical applications. Furthermore, in order to adjust their properties, introduction of necessary functional groups such as targeting moieties, and degradable units, these polymers need to be suitable for modification. In this sense, polyglycerol (PG, also known as polyglycidol), which is highly biocompatible and possesses all the mentioned requirements, has been thoroughly studied as an important polymer candidate in biomedical fields [6,7]. PG can be synthesized with different architectures via cationic, anionic, or coordination ring-opening polymerization of glycidol. Polyglycerol is highly water-soluble and biocompatible, and the hydroxy groups on each repeating unit enable numerous subsequent modifications [8].

In this review, we highlight the most common synthetic routes for obtaining polyglycerol with different architectures and highlight their application as carriers for active ingredients in different biomedical and pharmaceutical applications. A comprehensive and detailed overview of different polymerization methods of epoxide monomers has already been published elsewhere [9].

## 2. Synthetic Strategies for Polyglycidols

### 2.1. Cationic Ring-Opening Polymerization

#### 2.1.1. Hyperbranched Polyglycidol

**Figure 1:**
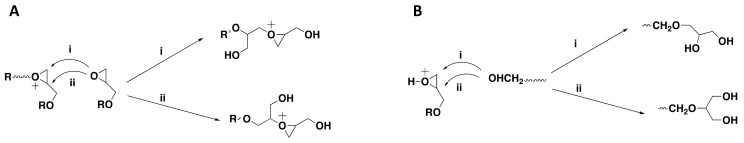
Proposed mechanism for CROP of glycidol based on (**A**) ACE and (**B**) AM. Adapted with permission from ref. [15]. Copyright 2017 Royal Society of Chemistry.

Cationic ring-opening polymerization (CROP) of heterocycles, which exhibits oxygen, is of high importance in the industrial production of engineering plastics such as poly(oxymethylene). However, due to a process called “back-biting”, which is intramolecular chain transfer, high amounts of cyclic byproducts can be formed. The first polymerization of glycidol was reported in 1966 when Sandler and Berg polymerized the AB_2_ type monomer (the theoretical representation suggested by Flory [10]) at room temperature using various catalysts. After that, Dworak et al., suggested, for the first time, the ring-opening polymerization of glycidol through a cationic route [11]. They proposed two possible mechanisms, namely active chain (AC) and activated monomer (AM) (Figure 1) [11]. The typical initiators for cationic ring-opening polymerization of glycidol were Lewis acids (BF_3_OEt_2_ or SnCl_4_) or Brønsted acids (CF_3_COOH or CF_3_SO_3_H) [11,12]. However, the main challenge was controlling the reaction kinetics since multiple side reactions can occur, which hinders propagation. Most HPGs synthesized following this route resulted in low molecular weights (less than 10,000 Da) and broad dispersities [11]. Nevertheless, the ease and simplicity of this method still engages researchers to explore possible ways to employ cationic ring-opening polymerization in investigating new HPGs architectures. Mohammadifar et al., reported a green synthesis route for synthesizing HPG using citric acid as an initiator [13]. They proved that not only the citric acid can initiate the propagating chain, but it is also incorporated into the polymeric backbone as it acts as a proton donor [13]. They suggested that the mechanism is based on AM cationic ring-opening polymerization [13]. With the citric acid molecule incorporated in the backbone, the HPG was degradable under neutral and acidic pH [13]. Although the polymerization did not lead to molecular weights higher than 1.5 kDa, the complete green synthesis and impuritiy-free polymers meant that it qualified as a good candidate for biomedical applications [13]. Recently, Kim et al., showed a recyclable metal-free catalytic system for the cationic ring-opening polymerization of glycidol also under ambient conditions using tris(pentafluorophenyl)borane as a catalyst [14]. The further propagation of polymerization, in this case, induced the precipitation of the higher molecular weight (1–4 kDa), highly hydrophilic HPG in nonpolar solvents, allowing the recycling of the catalyst and the solvent by the simple sequence of decantation of HPG [14].

#### 2.1.2. Linear Polyglycidol

Due to the above-mentioned side-reactions, CROP is not frequently applied for the polymerization of propylene oxide (PO) or similar compounds such as ethoxyethyl glycidyl ether (EEGE). The active growing chain-end by CROP is typically a secondary or tertiary oxonium cation, which can follow two discussed mechanisms: (i) ACE and nucleophilic attack of the oxygen of a cyclic monomer to a carbon atom of the oxygen which was formally bearing the positive charge which leads to propagation and chain growth. (ii) The AM mechanism, which is conducted in the presence of alcohols and allows better control over molecular weight while suppressing the cyclization issue [9]. In this case, the positive charge or the active end is on the monomer, and the polymer chain is inactive or dormant, which effectively hinders the back-biting side-reaction. However, the suitable reaction condition is to keep the instant concentration of monomer low; this can be achieved by slowly adding the epoxide monomer to the reaction mixture, which leads to prolonged reaction times and exact process control. Taken together, although AM CROP is a suitable method for the synthesis of telechelic polymers, substituted epoxide monomers such as PO are mostly polymerized with other techniques which can have more control over molecular weight and end-group fidelity [9].

### 2.2. Anionic Ring-Opening Polymerization

#### 2.2.1. Dendritic and Hyperbranched Polyglycerol (dPG and HPG)

**Figure 2:**
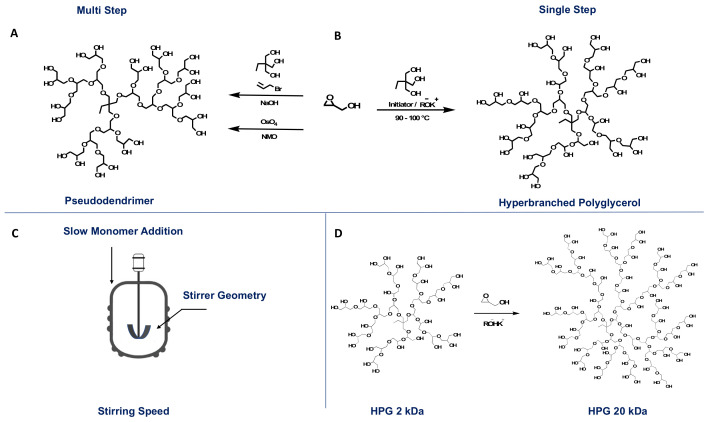
**Top:** (**A**) Pseudodendrimer, (**B**) HPG. **Bottom:** (**C**) reaction parameters, (**D**) (macromonomer, multistep).

A powerful single-step alternative to multistep polyglycerol dendrimers synthesis was reported by Haag and co-workers [16] (Figure 2a). Another method proposed by Sunder et al., combined anionic multi-branch ring-opening polymerization and slow addition of the monomer to obtain hyperbranched polyglycerol (Figure 2b) [17]. These two approaches overcome the limitations of the cationic ring-opening polymerization of glycidol. The initiator employed was a partially deprotonated 1,1,1-tris(hydroxymethyl)propane (TMP), and the reaction was carried out at 90–100 °C. The slow addition of glycidol ensured the controlled propagation of the polymerization and avoided low molecular weight chains due to the limitation of the intra-cyclization of oligomers. The resulting polymer was a medium-sized HPG with a relatively narrow dispersity (Đ < 1.5) [17]. However, even if the polymerization method reported by Sunder et al., demonstrated a controlled propagation and an end-polymer, the challenge still remains with reaching molecular weights higher than 6500 Da since the polymerization is solvent-free and the increase in viscosity will eventually result in poor mixing in the system [15,17]. Hence, synthesizing a high molecular weight polyglycerol is essential, especially for drug delivery system applications. High molecular weight will ensure that HPG possesses a large hydrodynamic size, enhanced vascular retention, and the presence of an extensive number of functional groups [15]. Schmitt et al., showed the dependency of the blood circulation of nanocarriers on HPGs molecular weight [18]. They tested various molecular weight HPGs nanocarriers (25–500 kDa) covalently linked to chelator desferrioxamine (DFO) and radiolabeled with the gamma emitter ^67^Ga [18]. Using qSPECT/CT imagining inside the heart of Rag2m mice, they proved that the blood circulation half-lives of the ^67^Ga labeled HPGs increase from 9.9 to 47.8 h with increasing molecular weight [18]. Different approaches have been suggested to synthesize high molecular weight HPGs. These approaches were well described in a review written by Kizhakkedathu’s group [15] that we would like to summarize in this review and describe other most recent methods. The first approach was reported by Frey and co-workers, which included the modification of stirring intensity, stirrer geometry, and monomer addition rate, as well as the addition of an inert emulsifying agent. This adjustment to the reaction parameters resulted in an HPG with a molecular weight of up to 20 kDa [19]. Recently, Haag’s group reported on the automated solvent-free polymerization of HPGs that ensures high reproducibility and traceability in the system due to automation [20]. The group reported a linear correlation between the torque and the degree of polymerization that can be applied to monitor the molecular weight during the polymerization [20].

The second approach is the macroinitiator approach, in which a reaction starts with the slow addition of glycidol to a partially deprotonated low molecular weight HPG acting as a macroinitiator. Wilms et al., employed a 500 and 1000 Da HPG-macroinitiator and reached a 24 kDa HPG [21], whereas Moore et al., started with a larger HPG-macroinitiator (2 kDa), which resulted in high molecular weight HPGs up to 100 kDa [22]. The third approach introduces a solvent as an emulsifying agent as another reaction parameter. Different solvents were screened, including dioxane [23], 1,4- dioxane, tetrahydropyran, ethylene glycol diethyl ether, and decane [24]. It was demonstrated that the use of the solvent does not hinder the properties nor the degree of branching of the resulting HPG [23]. In addition, the type of solvent can affect the exchange of counter cations with the propagating species, which can further affect the end-molecular weight [24]. Most recently, Kizhakkedathu’s group reported a gram-scale synthesis of mega hyperbranched polyglycerols (mega HPGs) with molecular weights in million Daltons up to 9.3 MDa and a Đ as narrow as 1.2 [25]. Kizhakkedathu’s group combined both the macroinitiator and the solvent-based approaches in the ROMP reaction. The macroinitiator was a partially deprotonated (10%) 840 kDa using KH and dissolved in DMF, where the slow monomer addition of glycidol was also followed to generate mega HPGs. The macroinitiator itself was synthesized using partially deprotonated TMP in dioxane at 90 °C with the slow addition of glycidol [25]. The mega HPGs conserved their properties of high water solubility, low intrinsic viscosity, and globular structure [25].

For HPGs to serve as a functional platform in biomedical applications and as drug delivery systems, the biodegradability aspect needs to be integrated into the HPG structure. Since it was proven that HPGs tend to accumulate in organs such as the liver in relatively high doses (10% of 500 kg/mol of the injected HPG) [26], For this purpose, the incorporation of physiologically degradable moieties into the HPG backbone will ensure the breakage of the structure when encountering the corresponding stimulus. Kizhakkedathu’s group incorporated ketal degradable linkage in the dendritic polyether backbone of HPG [27]. The polymer was synthesized following the anionic ROMP approach, using glycidol and the comonomer 2-(1-(2-(oxiran-2-ylmethoxy)ethoxy)cyclohexyloxy)ethanol) [27]. The ketal moieties were proven to degrade under mildly acidic conditions both in solution and within the cells [27]. Since the tumor site is characterized by a more acidic pH when compared to healthy cells, systems that are pH-responsive can serve as tumor-targeting drug delivery systems [28]. Kim’s group presented the anionic ROMP of a glycidol derivative molecule bearing a disulfide bond, i.e., 2-((2-(oxiran-2-ylmethoxy)ethyl)disulfanyl)ethan-1-ol (SSG) [29]. The polymerization resulted in a redox-degradable HPG. For an alternative synthetic approach, see chapter 2.3.1. The polymer was proved to be redox-responsive when treated with reducing agents, conserving the properties of HPG [29]. The cytosol of cancerous cells is characterized by an abundance of glutathione (GSH), 10 mM in comparison with <10 µM in healthy blood vessels and extracellular fluids; hence, a reductive sensitive drug delivery system is one of the approaches used to deliver active pharmaceutical ingredients (API) to the tumor site [30,31].

#### 2.2.2. Linear Polyglycidol

By chemical protection of the hydroxy group in glycidol, the proton exchange during polymerization can be efficiently hampered to obtain linear structures. In the next step after polymerization, deprotection yields linear polyglycerol (LPG) with free hydroxy groups. This strategy enables a toolbox for the design of various compositions and architectures using a myriad of protected glycidol monomers [7,32,33]. The multi-functional backbone of LPG allows a wide range of substitution reactions, resulting in high degrees of functionalization [32].

**Figure 3:**
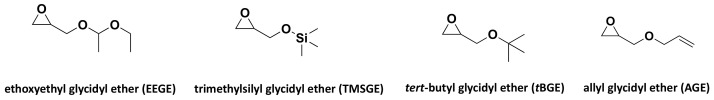
Common protected glycidols for synthesis of linear polyglycerol LPG. Adapted with permission from ref. [7]. Copyright 2014 American Chemical Society.

**Figure 4:**
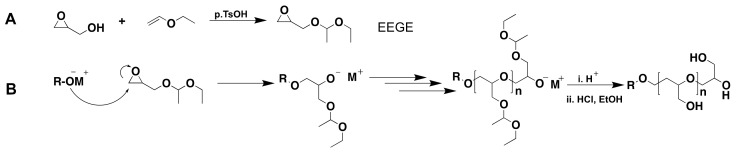
(**A**) Protection of glycidol, (**B**) polymerization of EEGE, and acidic deprotection of acetal groups. Adapted with permission from refs. [36,37]. Copyright 2010 American Chemical Society; 2021 Wiley Online Library.

LPG can be synthesized from glycidyl ethers, protected glycidol derivatives, by anionic ring-opening polymerization (AROP) [9], the classical way to polymerize epoxides to obtain polyethers and the main polymerization strategy since the 1930s [7,9]. Figure 3 represent the most common protected monomers applied for obtaining and preparing LPG.

tert-butyl glycidyl ether (tBGE) and allyl glycidyl ether (AGE) are commercially available. However, ethoxyethyl glycidyl ether (EEGE) is the most frequently used protected glycidol for LPG synthesis owing to its simple synthesis procedure and acidic deprotection of the protecting acetal groups [7,33]. EEGE was first synthesized by Fitton et al., in 1987 [34] and is prepared by the reaction of ethyl vinyl ether with glycidol in the presence of *p-*toluenesulfonic acid as a catalyst (Figure 4) [35,36].

In 1994 Taton et al., reported for the first time the successful polymerization of EEGE by applying cesium hydroxide as an initiator in bulk polymerization. The reaction resulted in poly(ethoxyethyl glycidyl ether) (PEEGE) with a molecular weight in the range of 30 kDa and a relatively broad dispersity (PDI: 1.5) [35].

**Figure 5:**
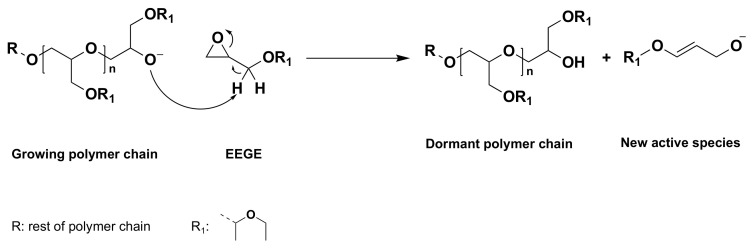
Possible mechanism of chain transfer reactions during the anionic polymerization of EEGE using alkali metal alkoxide initiators. Adapted with permission from ref. [40]. Copyright 2009 Elsevier.

Changing the initiator to potassium or cesium alkoxide resulted in the synthesis of polymers with narrow molecular weight distribution [38]. Until now, several initiators including potassium *tert*-Butoxide (*t-*BuOK) [39,40], potassium 3-phenyl propanolate (PPOK) [41,42], alkoxy ethanolates [43], potassium methoxide (MeOK) [44], and BuLi/phospahezene base (Li^+^/*t-*BuP_4_) [40,45] have been successfully applied for the controlled polymerization of EEGE. Nevertheless, for the polymerization of EEGE, alkali metal-based initiators lead to molecular weights limited to a maximum of about 30 kDa or a degree of polymerization (DP_n_) of about 300 [36,46]. Möller et al., explained this to be due to the chain transfer reaction to a monomer either from the active chain-end or the oxyanion initiator [40]. Other monosubstituted epoxides such as PO or phenyl glycidyl ether are reported to undergo this chain transfer in the same way during the polymerization [47]. The proton substitution from the adjacent group to the epoxide ring results in the formation of an unsaturated allyl alkoxid. (Figure 5) [40].

With the increase in temperature and in higher monomer to initiator ratios, this transfer reaction was more noticeable. In order to obtain polyethers with high molecular weights, it is generally assumed that the nucleophilicity of the active propagating chain-end has to be high enough to utilize the ring-opening of the epoxide ring but have relatively low basicity to prevent proton transfer and side-reactions [7].

### 2.3. Coordination Ring-Opening Polymerization

#### 2.3.1. Hyperbranched Polyglycidol

Harth et al. developed an alternative method to synthesize semi-branched polyglycidols to the traditional ionic polymerizations. They investigated the homopolymerization of glycidol by employing Tin(II) trifluoromothenesulfate (Sn(OTf)_2_) as a catalyst and varying the temperature. The results showed control over the branching of the polyglycerol backbone by varying the temperature and creating different protein-glycidol bioconjugates as an alternative to pegylated biostructures [48].

**Figure 6:**
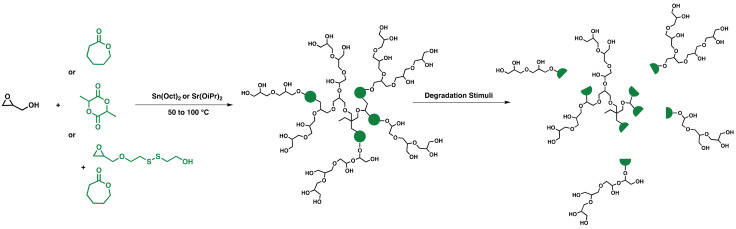
Synthesis of degradable HPG via coordination–insertion mechanism. The degradation follows different stimuli such as changes in pH, reductive environment, etc.

The bulk polymerization of cyclic esters using a catalyst such as Tin(II) 2-ethylhexanoate (Sn(Oct)_2_) to form polyesters has been proven to be controlled and pseudoliving [49]. Additionally, given the high epoxide ring strain of the glycidol monomer, a new strategy has emerged to synthesize biodegradable high molecular weight HPGs by copolymerizing cyclic esters and glycidol following the catalytic route and a coordination-insertion ring-opening polymerization mechanism. Haag’s group was dedicated to synthesizing and investigating such systems in solvent-free one-pot synthesis. Figure 6 shows some of the strategies to obtain degradable HPG. Cherri et al., proved the scalability and reproducibility of the sulfated HPGs bearing bio-degradable caprolactone segments system with controlled molecular weights (20–60 kDa) in two-step synthesis [50]. The system showed the ability to encapsulate the hydrophobic tyrosine kinase inhibitor chemotherapeutical drug sunitinib [50]. The drug delivery system was able to release its guest molecule under acidic and enzymatic conditions, accumulate in the tumor site, and perform better than the free sunitinib in vivo [50].

Another system developed by Zabihi et al., followed a similar strategy to synthesize poly(glycidol-lactide) up to 43 kDa [51]. Further, the system was then loaded with tacrolimus (TAC), a macrolide immunosuppressant which is used for the treatment of atopic dermatitis [51]. TAC has low bioavailability, and the system was proven to load TAC effectively (14.5% *w*/*w* loading capacity) and ensure its educated delivery into the stratum corneum, viable epidermis, and upper epidermis when compared with Protopic^®®^ (containing 0.03% *w*/*w* of TAC) [51]. On the other hand, Reisbeck et al., developed an HPG sulfates drug delivery system that was dual degradable based on the copolymerization of glycidol, ε-caprolactone, and SSG, the glycidol derivative monomer bearing a disulfide bond in bulk screening different catalysts [52]. The catalyst that was most prominent for polymerization was strontium isopropoxide, leading to the highest molecular weight and degree of branching [52]. The system was degradable under enzymatic and reductive stimuli and was capable of encapsulating and releasing doxorubicin under the same conditions [52].

#### 2.3.2. High Molecular Weight Linear Polyglycidol

To obtain high molecular weight LPG, coordination-type polymerizations using organometallic catalysts were also conducted using EEGE as a monomer [53,54,55].

Haout et al. prepared LPG with high molecular weights of about 1 kDa by applying diethylzinc and water as an initiating system. However, poor control over the molecular weight leads to higher dispersities (PDI = 1.46–1.80) [54].

**Figure 7:**
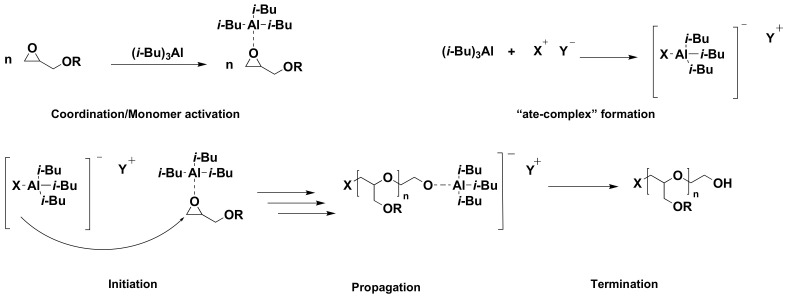
Reaction mechanism of activated monomer polymerization technique. Adapted with permission from ref. [9]. Copyright 2016 American Chemical Society.

In 2007, using a dual initiating system composed of tetraoctylammonium bromide (Oct)_4_NBr and triisobutylaluminium (*i*-Bu)_3_Al, Carlotti and Deffieux et al. reported a polymerization strategy, which was a breakthrough for the polymerization of many functional epoxides including EEEGE [56]. This strategy is based on parallel activation of the monomer towards nucleophiles while reducing the basicity of the growing chain-end through coordination with the Lewis acid (catalyst). The coordination of the catalyst with the epoxide ring results in the reduction of electron density in the ring and hence promoting ring-opening (monomer activation). At the same time, the catalyst and the initiating species (a weak nucleophile) form an “ate-complex”, through which the initiation begins (Figure 7) [9]. This polymerization offers the advantage that the polymerization can be performed at lower temperatures (from −30 °C to R.T), and the chain transfer reactions of adjacent proton to epoxide ring are effectively suppressed, especially in systems where ammonium salts and (*i*-Bu)_3_ Al are combined [9]. Gervais et al., further applied this system to prepare PEEGE to molecular weights up to 85 kDa with narrow molecular weight distributions (PDI ≥ 1.03) [36]. Although side reactions such as ring-opening via hydride or *iso*-butyl groups can cause lower molecular weights and ill-defined chain-ends, for the preparation of high molecular weight polyethers with defined structures, this “activated monomer” polymerization is a widely studied and applied method in comparison to the conventional AROP [9,57,58]. In a study aiming toward the synthesis of biodegradable LPG, Köhler et al., synthesized polyglycidol with graftings of polycaprolactone [59].

## 3. Polyglycidols as a Multiplatform for Biomedical Applications

### 3.1. Drug Delivery Systems, Protein and Surface Conjugation

HPG is characterized by its high water-solubility and end group tune-functionality. Hence, its derivatives can be designed and functionalized to be used as a drug delivery platform when modified to include biodegradability to avoid in vivo accumulation and release its cargo and hydrophobicity to encapsulate hydrophobic cargos as additional properties. Nevertheless, HPG can also be integrated into other supramolecular structures such as polymeric micelles or nanogels to form drug delivery systems. As HPG-based drug delivery systems have been intensively discussed in previous reviews [15], we would like to summarize the most recent systems in the scope of this review.

Zhong et al. developed amphiphilic block copolymers that included sulfated HPG as its hydrophilic part, attached with a disulfide-bearing linker to polycaprolactone as the hydrophobic part [60]. The amphiphilic block copolymer underwent self-assembly to form micellar structures. The micellar system was then loaded with doxorubicin (DOX) and proved to have a long plasma half-life and significant tumor accumulation [60]. When tested on MCF-7 human mammary carcinoma mice models, the micelles induced complete tumor suppression and improved the survival rate [60]. Later, Braatz et al., assembled a toolbox of the same micellar system that contained various polyesters as the hydrophobic segment as well as two different molecular weights of the sulfated HPG hydrophilic segment [61]. The study also included determining the critical micelle concentration (CMC), testing the stability, drug release, and tumor targeting of the system when encapsulated with sunitinib [61]. When the loaded micelles were injected into an HeLa human cervical tumor-bearing mice model, a ten-fold lower dose of the micelle in comparison to the free drug was able to improve the antitumor efficacy of the API [61].

Baabur-Cohen et al. developed two polymeric carriers that have different supramolecular assembly architectures for the combination drug therapy of paclitaxel (PTX) and doxorubicin (DOX) [62]. The drugs were covalently bonded to the linear section of the polymer, polyglutamic acid (PGA), or to the hyperbranched scaffold of PG that was attached to polyethylene glycol (PEG), respectively, in both polymeric architectures [62]. The aim was to study the activity of both conjugates in a murine model of mammary adenocarcinoma in immunocompetent mice. In this regard, both conjugates showed superior antitumor activity when compared to the combination of the two free drugs [62]. Furthermore, researchers have investigated thermoresponsive nanogel (tNGs) drug delivery systems with HPGs platforms. One of the studies presented by Rancan et al., aimed to improve dermal and transdermal drug delivery using tNGs based on acrylated dendritic polyglycerol as a water-soluble crosslinker, combined with ethylene glycol methacrylate with thermoresponsive properties [63]. The characteristics of the tNGs were dependent on their cloud point temperature (Tcp), as well as their skin penetration and cellular uptake [63]. Gerecke et al., proved the intracellular localization of HPG-based tNGs, a combination of dPG with poly(glycidyl methyl ether-co-ethyl glycidyl ether), in keratinocytes [64]. The tNGs were able to encapsulate dexamethasone and tacrolimus, drugs used for skin disease treatment. By laser scanning confocal microscopy, it was proven that the fluorescently labeled system was able to localize predominantly within the lysosomal compartment [64]. In addition, the tNGs showed no cytotoxic or genotoxic effect, any induction, or reactive oxygen species when the MTT assay, comet assay, and carboxy-H2DCFDA assay were performed. Later, the same TNGs were tested for the dependency of the uptake mechanism on the cloud point temperature of the TNGs [65]. Intriguingly, it was shown that above the Tcp, the uptake mechanism was caveolae-mediated endocytosis; however, at the Tcp, micropinocytosis was also included as an uptake mechanism [65]. Other thermoresponsive nanogel carrier systems were developed for controlled delivery through the hair follicle by Sahle et al., The nanogels were synthesized by the precipitation polymerization technique using N-isopropylacrylamide as a monomer, acrylated dendritic polyglycerol as a crosslinker, VA-044 as an initiator, and sodium dodecyl sulphate as a stabilizer [66]. The follicular penetration of the labeled nanogels was assessed ex vivo using porcine ear skin [66]. Another TNGs was developed by Molina et al. and based on Poly-*N*-isopropylacrylamide–dendritic polyglycerol NG that was semi-interpenetrated with 2-acrylamido-2-methylpropane sulfonic acid or (2-dimethylamino) ethyl methacrylate [67]. The tNGs were loaded with doxorubicin and showed more efficiency in multidrug-resistant cancer cell proliferation inhibition studies. When admitted in vivo, the tNGs reduced the tumor volume to about 25% [67]. Miceli et al., combined the thermoresponsiveness with the pH responsiveness of the NGs based on dendritic polyglycerol (dPG) and pNIPAM that were semi-interpenetrated with poly(4-acryloylamine-4-(carboxyethyl)heptanodioic acid) (pABC) [68]. They showed the stability of the system under physiological conditions and tunable electrophoretic mobilities around the human body temperature [68]. The NGs were able to release their hosted molecule (cytochrome c) upon cooling and were able to deliver it to cancer cells and induce apoptosis at 30 °C [68].

Hofmann et al. developed polyethylene glycol (PEG)-substituted liposomes by developing multi-functional lipids based on the AROP of protected glycidols (EEGE and isopropylidene glyceryl glycidyl ether (IGG)) initiated by cholesterol and 1,2-bis-n-alkyl glyceryl ether. Due to the multi-functionality of these liposomes, they can be further functionalized. The authors used rhodamine B as an example for further modification [69]. For selective delivery and release of species for biomedical applications, Jamróz-Piegza et al., designed and synthesized deblock copolymers of PEG and LPG via AROP, where the LPG block was modified with cinnamic acid in a subsequent step [70]. After micelle formation, owing to the properties of glycidyl cinnamate groups, the core could be crosslinked by UV-irradiation, forming stable nanoparticles.

LPG was recently thoroughly studied as an alternative to PEG for the conjugation of biopharmaceuticals due to its structural similarity to PEG. In the last three decades, PEG has been the “gold standard” for conjugation to biopharmaceuticals (a process known as PEGylation) to address their intrinsic shortcomings such as instability, immunogenicity [71], and a short half-life [72,73]. To date, there are more than 18 PEGylated drugs approved by the Food and Drug Administration (FDA) on the market [74]. PEGylation can also be applied to lipids and nanoparticle (NP) drug delivery systems to enhance the stability of NPs [75]. This technology has been employed in the new mRNA vaccines against SARS-CoV-2 [76]. However, despite these major breakthroughs, there are some shortcomings associated with PEGylated systems which can limit their broad application. These include: induced anti-PEG antibodies upon repeated administration of PEGylated drugs, negatively altering their therapeutic efficiency and, in some cases, leading to life-threatening allergic reactions such as anaphylaxis [77]. Similar to PEG, LPG is based on a polyether backbone but exhibits hydroxy groups leading to much higher hydrophilicity of the polymer backbone; in contrast to conventional PEG, LPG enables the introduction of immobilization, targeting, or labeling moieties [78,79]. Additionally, esters of oligoglycerols with up to 10 repeating units have been approved by the FDA as pharmaceutical and food additives and have been commercially available for a few decades [7,32]. In a study comparing, LPG, hPG, and PEG, Imran Ul-haq et al., tested these polymers with molecular weights of about 100 kDa both in vitro and in vivo. They showed that the hydrodynamic size measurements confirmed the absence of intermolecular aggregation. It was observed that the intrinsic viscosity of LPG was about 25 times smaller than that of PEG of the same size. These characteristics play an important role in the application of these polymers in formulations where higher doses are needed. LPG was further tested in different hemocompatibility assays, such as red blood cell (RBC) aggregation and homolysis. They showed that LPG (and HPG), even in high concentrations (10 mg/mL), did not induce any unwanted RBC aggregation, while PEG induced a massive RBC aggregation at the same concentration [80]. This was in line with the result of this assay performed with lower molecular weight LPG (6400 g/mol) by Kainthan et al., [81]. Their hypothesis was that although LPG and PEG have similar structures, the more compact and hydrophilic structure of LPG limits its interaction with RBC. Furthermore, LPG did not induce any platelet or complement activation in the concentration range which was studied (up to 10 mg/mL). In contrast, a mild to moderate complement activation already at 5 mg/mL was induced by PEG. Furthermore, a longer half-life for LPG was observed in comparison to other polymers [80].

In a study conducted by Abu Lila et al. liposomes were modified with PEG and LPG. It was observed that replacing LPGylated liposomes enhances the in vivo performance in comparison to PEGylated counterparts, as LPG-modified liposomes did not induce accelerated blood clearance (ABC), a limitation PEGylated liposomes have upon repeated administration, which negatively affects their pharmaceutical activity [82]. The end-group of LPG can be easily modified to introduce the desired functional group for protein modification or other applications. This can be carried out either by applying different initiators or post-modification of the end group subsequent to the polymerization process [5,33,36,83,84,85]. LPG has been successfully conjugated to proteins via different chemistries, including random conjugation to bovine serum albumin (BSA) [33], site-specific CuAAC to Exenatide, *N*-terminally to Interleukin-4 [86], and Anakinra [87] and to IFN-α-2a via SPAAC [88]. These studies clearly demonstrated a comparable size of PEGylated and LPGylated proteins with a similar half-life extension in vivo. Owing to its excellent biocompatibility, ease of synthesis, multi-functionality, and low intrinsic viscosity, LPG is a promising candidate for various other biomedical applications.

### 3.2. Viral Infection Inhibition

Viral diseases are one of the major threats to our health, which are associated with morbidity, mortality, and serious socioeconomic consequences [89]. Vaccination, as the most effective solution, is only available against a limited number of viral infections [90]. Antiviral small molecule drugs are likewise effective against a limited number of viruses and target essential viral functions and proteins, meaning virus mutations can escape and make the virus resistant to drugs [91]. Therefore, the development of alternative ways to intervene with a broad spectrum of virus families is of high interest.

**Figure 8:**
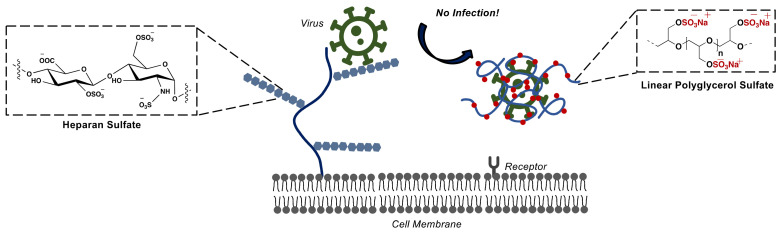
Competitive multivalent binding of a virus to a sulfated linear polyglycerol scaffold instead of heparan sulfate and infection inhibition.

A primary mechanism that several viruses have developed for binding and delivering the viral genome is initial multivalent binding to different heparan sulfate proteoglycans (HSPGs) at the host cell surface [92,93,94]. HSPGs are composed of protein cores covalently linked to unbranched negatively charged polysaccharides named heparan sulfate (HS) [95]. The counter ion release is the main driving force for these electrostatic interactions. The positively charged patches on the virus surface play the role of many counterions for the negatively charged HS. This interaction is favored entropically due to the release of the counterions from the polyelectrolyte. It has recently been shown that interaction with HS is a necessary co-factor for SARS-CoV-2 cell entry and infection. Inspired by HS, negatively charged natural polysaccharides such as heparin [96,97] are used as viral infection inhibitors. Some biological activities of heparin include angiogenesis and tumor growth inhibition [98,99], complement system regulation [100,101], and antiviral [97,102] and anti-inflammatory [103] activity. It is approved by FDA for clinical use in the treatment of deep vein thrombosis. Furthermore, all these properties of heparin make it a unique and promising treatment candidate for COVID-19 patients due to thromboembolic events [104] and pathological inflammation [96,105]. Recently it was shown that heparin can effectively inhibit SARS-CoV-2 infection. The application of heparin for the treatment of COVID-19 patients is under intense investigation, and nebulized heparin in the treatment of the SARS-CoV-2 infection has reached clinical trials [4,100,106,107]. However, currently, the only source of heparin is animal tissues which raises the risk of virus contaminations, adverse effects, and batch-to-batch variability [92,108]. Additionally, heparin can be altered or degraded by heparinases which could result in loss of its activity [100]. Another issue is the anticoagulant activity of heparin which can lead to undesired side effects such as hemorrhagic complexities [109]. Therefore, designing heparin-mimicking compounds to overcome the limitations is intensively studied. Our group recently introduced linear polyglycerol sulfates (LPGS) as heparin mimetics and powerful SARS-CoV-2 inhibitors (Figure 8). Surprisingly the LPGS with a molecular weight of 40 kDa was almost 60 times more active than heparin but showed a much lower anticoagulant activity [110].

Their biocompatibility and multi-functionality, and a plethora of different obtainable structures, make polyglycerols a promising platform for designing novel multivalent virus inhibitors. Bhatia et al., investigated the role of the flexibility of the scaffold in the inhibition activity in a systematic comparison between functionalized LPG and HPG with similar molecular weights for inhibition of IAV [111]. They observed that the linear backbone inhibited IAV more efficiently than the hyperbranched counterpart both in vitro and in vivo. They further investigated this observation in polyglycerol-based nanogel (NG) scaffolds [106]. NGs are cross-linked 3D constructs from water-soluble polymers and are swellable. For the introduction of different flexibilities into the gels, they applied LPG and HPG as flexible and rigid cross-linkers, respectively, to obtain flexible (F-NG) and rigid (R-NG) cross-linkers, and observed that the most flexible functionalized NG based on LPG can inhibit IAV infection 400 times better than the more rigid counterparts [106].

Dey et al. made this observation for the inhibition of HSV-1 by NGs. They prepared two classes of NGs based on sulfated HPG (HPGS) and used LPG or HPG as cross-linkers to develop scaffolds with distinctive rigidities. They further proved the flexibility differences between F-NGs and R-NGs by atomic force microscopy (AFlinearM), in which the negatively charged NGs were coated on a positively charged mica substrate, and it was observed that the F- NG, which was made with LPG linker, exhibited a smaller height and higher width in comparison to R-NG cross-linked with HPG. It was additionally observed that flexible NG had increased antiviral activity due to better shielding of the virus interaction with the cell surface. Their mathematical modeling supported this data by showing that to sterically shield a virus particle, six rigid NG are needed while only three of the soft NG of the same size are needed [107]. Pouyan et al. investigated the effect of backbone flexibility on the inhibition of HSV-1 by synthesizing a series of sulfated polyglycerols with different architectures, namely linear, dendronized, and hyperbranched (LPGS, DenPGS, HPGS) and compared it to Heparin as the natural sulfated polymer [112]. From the plaque reduction assay, an increase in IC_50_ values from 0.03 to 374 nM from the flexible backbone (LPGS) to less flexible ones (dendronized and HPGS) was observed. Knowing that all the polymers had the same density of negative charges, it was concluded that the more flexible the backbone, the more it can change conformation and shield the virus surface. To further evaluate the role of the scaffold’s flexibility, Mohammadifar and Ahmadi et al. used HPGS to form 2D constructs by reversibly fixing HPG on a graphene sheet, crosslinking them together to form a 2D construct, separation from graphene sheet, followed by sulfation. This system was then compared with sulfated 3D NGs in viral infection inhibition. Due to higher flexibility, the 2D system outperformed the 3D system by showing four times better inhibition of HSV-1 and SARS-CoV-2 [113].

To investigate other heparin-like characteristics of the HPGS, Ferraro and Silberreis et al. investigated the anti-inflammatory properties of this scaffold. They observed that HPGS has anti-inflammatory activity and can regulate the complement response as good as heparin [101], and much less anticoagulation time for this material was detected, which is of high interest for applications of anti-inflammatory or antiviral compounds to reduce the risk of uncontrolled bleedings.

### 3.3. Antifouling Coatings for Biomedical Application

**Figure 9:**
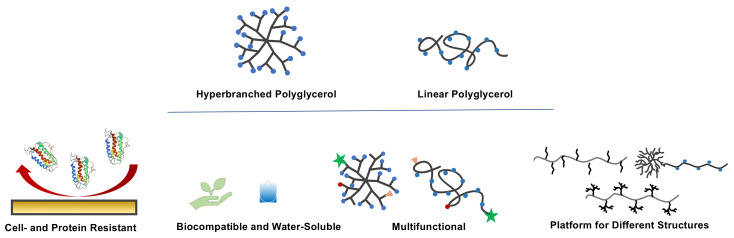
Properties of polyglycerols make them suitable candidates for biomedical applications.

Unspecific biofouling or nonspecific protein adsorption on surfaces present a serious problem in biomedical applications such as medical implants, biosensors, and surgical equipment [114]. Due to its antifouling characteristics, PEG has been the focus of many studies for the development of non-fouling coatings for biomedical applications [115,116]. As PEG-modified surfaces have disadvantages such as the instability of the polymer upon heating and immunological challenges after repeated exposure [117], LPG has been investigated as an alternative to PEG for surface modification. Kulka et al., developed antifouling surface coatings based on mussel-inspired dendritic PG (MI-dPG) modified with LPG containing a block of oligo-amine (LPG-b-OA_11_) and compared the surface characteristics such as cell fouling, protein fouling, and chemical stability with the MI-dPG surface modified with commercially available amine terminated PEG (HO-PEG-NH_2_). The quartz crystal microbalance measurements with dissipation monitoring (QCM-D) revealed that the post functionalized surfaces with LPG outperformed the PEG modified surfaces in protein antifouling properties. In a follow-up study, the authors investigated the applicability of these coatings in regard to reducing shear and biomaterial-induced thrombosis on continuous-flow ventricular assist devices [118]. They observed that the post-modified surfaces with LPG outperformed PEG-modified surfaces in rejection of cellular adhesion and proliferation. With regard to cell toxicity, the LPG-b-OA_11_ showed no cytotoxicity up until 5 mg^.^mL^−1^ on A549 cell lines. In another study attempting to develop antifouling surfaces based on PG, the authors reported a simple and solvent-free surface-initiated polymerization from MI-dPG-coated TiO_2_ (hydrophilic) and polydimethylsiloxane (PDMS, hydrophobic) [119]. They performed cell viability studies with two different cell lines (A549 and DF-1) on various coatings to observe the influence of the coating on the cell numbers. The results showed that the introduction of MI-dPG on both TiO_2_ and PDMS led to a slight decrease in the cell number on the respective surface. However, after grafting dPG, a drastic decrease (>95%) in the cell number was observed for both cell lines and investigated surfaces. Their approach provided a successful strategy for developing a highly biocompatible but cell-repelling surface coating [119]. Figure 9 represents an overview of the properties of PG discussed in this and previous sections.

## 4. Summary

Functional polymers are an indispensable tool in medicine and life sciences. PG, with different structures and properties such as water-solubility, biocompatibility, and multi-functionality, has become one of the most studied and applied polymers in these fields (Figure 9). In the past few decades, great efforts have been invested to optimize the synthesis and modification of these polymers for biomedical and pharmaceutical applications. HPG can be synthesized via cationic, anionic, or coordination ring-opening polymerizations from the monomer glycidol. By protecting the hydroxy group of the monomer prior to polymerization, linear structures (LPG) are obtained. The most common monomer for this purpose is EEGE, in which the hydroxy group of glycidol is protected by an acid-labile acetal group.

PGs have been successfully applied in targeted drug delivery systems as carriers and micelles. Furthermore, moieties with redox or pH-sensitive groups have been successfully introduced to the backbone, making these systems degradable under biological conditions. Due to similar characteristics of LPG to the gold standard PEG, it has been conjugated to biomacromolecules such as proteins to increase their stability, solubility, and half-life.

We believe that the characteristics, ease of synthesis, scalability, multi-functionality, and structural versatility of PGs and PG-based systems can make a significant impact on the development and application of these materials both in vitro and in vivo.
